# Association between red blood cell folate and accelerated aging in American adults: a cross-sectional study from the national health and nutrition examination survey

**DOI:** 10.3389/fnut.2025.1504441

**Published:** 2025-06-19

**Authors:** Jia-ni Wang, Zhen Song, Cheng Xu, Chong-chao Li

**Affiliations:** ^1^Institute of Literature in Chinese Medicine, Nanjing University of Chinese Medicine, Nanjing, China; ^2^Yancheng Binhai Hospital of Traditional Chinese Medicine, Yancheng, China; ^3^The First Clinical Medical College, Nanjing University of Chinese Medicine, Nanjing, China

**Keywords:** RBC folate, phenotypic age acceleration, biological aging, NHANES, U-shaped relationship

## Abstract

**Objective:**

The study aims to explore the relationship between red blood cell (RBC) folate concentrations and accelerated aging.

**Methods:**

Data were derived from the National Health and Nutrition Examination Survey (NHANES) cycles of 2007–2010, including 8,944 participants aged ≥ 20 years. Phenotypic age acceleration (PhenoAgeAccel) was calculated using chronological age and 9 aging-related biomarkers. Multivariate linear regression and generalized additive models were used to analyze the relationship between RBC folate levels and PhenoAgeAccel. Smooth curve fitting was used to explore the potential non-linear relationship and threshold effect analysis was applied to examine inflection point.

**Results:**

The analysis revealed a U-shaped relationship between RBC folate levels and PhenoAgeAccel, with the inflection point at 732.9 ng/mL. The PhenoAgeAccel decreased by 0.0027 years per 1 ng/mL increase in RBC folate when RBC folate ≤ 732.9 ng/mL (β: −0.0027, 95% CI: −0.0051, −0.0002), and increased by 0.0058 years per 1 ng/mL increase in RBC folate when RBC folate > 732.9 ng/mL (β: 0.0058, 95% CI: 0.0026, 0.0090). Subgroup analysis indicated consistent associations across most demographic and health categories, except for a positive correlation in participants with cardiovascular diseases.

**Conclusion:**

There was a U-shaped association between RBC folate and accelerated aging among US adults.

## 1 Introduction

By 2030, one-sixth of the global population is projected to be aged 60 or over (1). However, the increase in lifespan has not matched by a corresponding increase in the length of the healthy lifespan, indicating that the additional years are not necessarily spent in good health (2). There is growing recognition that promoting healthy aging is more important than merely preventing death. By reducing the incidence and progression of aging-related diseases such as heart disease, loss of function, and cognitive decline, even a longer life expectancy can be achieved by slowing the aging process (3, 4).

Despite the chronological age is an important factor in the development of aging-related diseases and mortality, it does not accurately represent biological aging. Phenotypic Age (PhenoAge) is a quantifiable aging indicator that has been demonstrated to be more effective in identifying aging-related disease risk and mortality than previously proposed indicators, such as telomere length, DNA methylation age, and serum Klotho concentration (5, 6). PhenoAgeAccel, the residual from regressing PhenoAge on chronological age, represents whether a person is physically younger or older than their chronological age (7).

Nutritional interventions, which can potentially reduce aging-related disease risk and promote longevity, have gained significant research interest (8). Among the 12 hallmarks of aging (9), available evidence suggests that folate is associated with markers of DNA instability, telomere attrition, epigenetic alterations, mitochondrial dysfunction, cellular senescence, and chronic inflammation (10–14). Furthermore, under folate fortification policies, potential risks of a high-folate states have come into public view (15). However, previous studies have raised concerns about the potential risks of excessive folate intake, such as masking vitamin B12 deficiency, promoting the progression of certain cancers, and increasing the risk of cognitive decline in the elderly (16). Studies on folate primarily focus on its relationship with aging mechanisms and diseases, and the definition of high levels of folate remains controversial. On the other hand, folate deficiency remains a public health issue in some populations, particularly in low-income countries and among specific vulnerable groups (17). Therefore, it may be necessary to determine the optimal folate intake levels across populations to guide the development of more targeted and effective public health strategies.

To fill these knowledge gaps, we aimed to explore the relationship between RBC folate concentrations and accelerated aging among adults in the US population, to provide insights into slowing aging process.

## 2 Materials and methods

### 2.1 Study population

**FIGURE 1:**
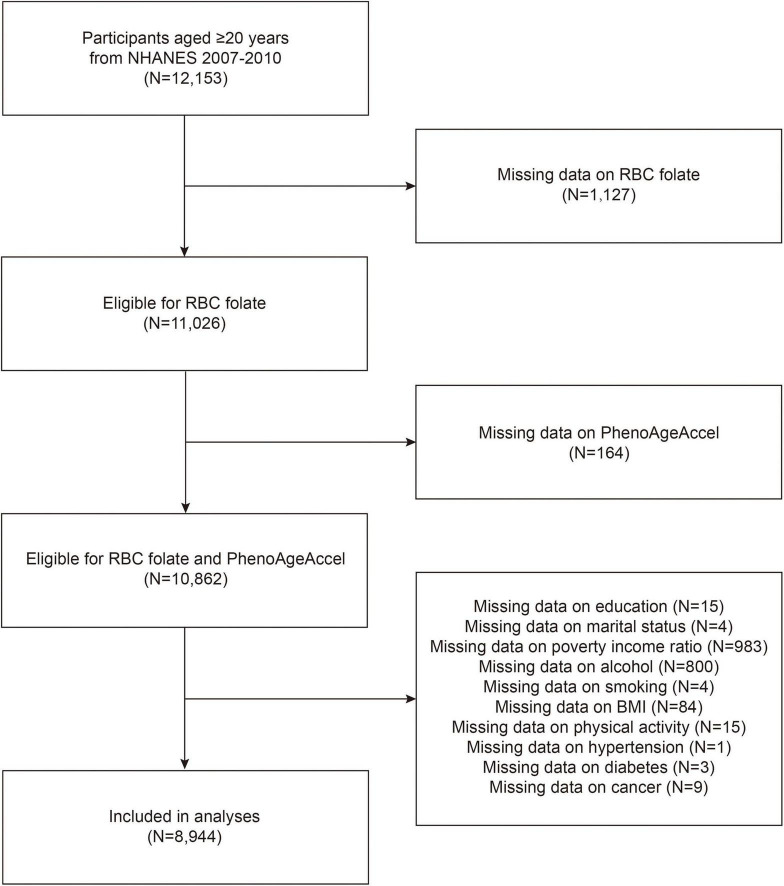
Flow chart of participants selection. NHANES, National Health and Nutrition Examination Survey; RBC, red blood cell; PhenoAgeAccel, phenotypic age acceleration; BMI, body mass index.

The National Health and Nutrition Examination Survey (NHANES) is a cross-sectional survey designed to assess the health and nutritional status of a nationally representative sample of the US civilian population.^1^ The survey includes questionnaire interviews, laboratory data and physiological examinations. Considering the availability of RBC folate and PhenoAge data, this study utilized data from two NHANES cycles (2007–2010), including 12,153 participants aged 20 years and older. Participants with missing data for RBC folate (*N* = 1,127) and those lacking biomarkers for the PhenoAgeAccel algorithm (*N* = 164) were excluded. The final sample size was 8,944 individuals, following the exclusion of participants lacking other covariates (*N* = 1,918) (Figure 1).

### 2.2 Measurement of RBC folate

RBC folate is a classical biomarker of folate status. The European Food Safety Authority (EFSA) considers RBC folate concentration to be the most reliable indicator of folate status (18). Whole blood and serum specimens were collected and then stored at ≤ −20°C until transported to the National Center for Environmental Health for analysis (specimens should be frozen at −70°C for long-term storage). RBC folate concentrations were measured using the microbiologic assay (MA) (19).

### 2.3 Measurement of PhenoAgeAccel

PhenoAge and PhenoAgeAccel are specifically quantifiable and observable aging indicators developed based on NHANES data to estimate individuals at high risk for multiple diseases and all-cause and disease-specific mortality. The PhenoAge algorithm was developed on chronological age and nine biomarkers from the NHANES III dataset: albumin, creatinine, glucose, C-reactive protein, white blood cell count, lymphocyte percent, red blood cell distribution width, mean cell volume and alkaline phosphatase (7). PhenoAgeAccel was calculated as a residual from a linear regression of PhenoAge against chronological age, a negative PhenoAgeAccel value represents a person who is physiologically younger than their chronological age, while a positive PhenoAgeAccel value indicates a person who is physiologically older (20). The detailed calculations of PhenoAge and PhenoAgeAccel are presented in Supplementary Methods 1.

### 2.4 Covariables

NHANES collected the following covariates through standardized questionnaires. Sociodemographic factors including age, sex, race (Mexican American, other Hispanic, non-Hispanic White, non-Hispanic Black, other race), education level (< high school, high school, > high school), marital status (married/living with partner, widowed/divorced/separated, never married), ratio of family income to poverty (PIR). The PIR reflects a household’s income relative to federally defined poverty thresholds, calculated by dividing total income by guidelines adjusted for family size, state, and year. Smoking status was categorized as current smokers, formerly smoked, and never smoked. Body Mass Index (BMI) was calculated as measured weight (kg) divided by height squared (m^2^) and categorized by 25 and 30 kg/m^2^. Alcohol consumption was categorized based on whether participants consumed at least 12 alcoholic drinks per year. Physical activity was classified as inactive (participants who did not engage in either vigorous or moderate physical activity), moderate (activity done for at least 10 min that caused only light sweating or a slight to moderate increase in breathing or heart rate), or vigorous (activity done for at least 10 min in the past 30 days that caused heavy sweating or large increases in breathing or heart rate). The health condition data were composed of hypertension, diabetes, cardiovascular disease and cancer. Hypertension was defined as a prior diagnosis of hypertension, current use of antihypertensive medications, or having a systolic blood pressure level of ≥ 130 mmHg and/or a diastolic blood pressure level of ≥ 80 mmHg (21). Diabetes was defined as a prior diagnosis of diabetes, current use of insulin or diabetes pills, fasting plasma glucose level ≥ 126 mg/dL, or a hemoglobin A1c level ≥ 6.5% (22). Cardiovascular disease and cancer were assessed by self-reported questionnaires.

### 2.5 Statistical analysis

Based on the NHANES analytic guidelines, appropriate sampling weights were applied to interpret the complexity of survey design in NHANES database during our analysis. The baseline characteristics of the participants included were described using weighted means for the continuous variables or proportions for the categorical variables. One-way ANOVA or Kruskal–Wallis and the χ2 test were conducted to compare the differences between groups. A multivariate linear regression model was used to assess the linear relationship between RBC folate levels and PhenoAgeAccel. Three models were constructed. Model 1 did not adjust for any covariates, while Model 2 was adjusted for age, gender and race. Model 3 further adjusted for education, marital status, poverty income ratio, BMI, alcohol, smoking, physical activity, hypertension, diabetes, cardiovascular disease, and cancer. In addition, generalized additive models and smoothed curve fits were used to examine non-linear relationship between RBC folate and PhenoAgeAccel. Recursive algorithms and two-stage logistic models were utilized to detect any potential inflection points in the relationship. Moreover, subgroup analyses and interaction tests were conducted to explore whether the associations differed across subgroups defined by age, gender, BMI, hypertension, diabetes, cancer, and cardiovascular disease (23). All the analysis were performed with R (version 4.3.1)^2^ and EmpowerStats (version 4.2).^3^ *P* < 0.05 was considered statistically significant.

## 3 Results

### 3.1 Participant characteristics

The baseline characteristics of the studied variables across tertiles of RBC folate are presented in Table 1. Our analytical sample included 8,944 participants aged ≥ 20 years. The mean age was 46.85 years, and 50.93% of participants were female. Participants with high RBC folate levels were more likely to be female, non-Hispanic White, have a higher level of education, be married or living with a partner, consume higher amounts of alcohol, never smoked and have lower PhenoAgeAccel value. Additionally, compared to the low RBC folate level group, participants with the high RBC folate levels suffer from hypertension, diabetes, cardiovascular disease and cancer.

**TABLE 1:** Basic characteristics of the study participants.

Characteristics	RBC folate			*P*-value
	Low	Moderate	High	
Age (years)	42.55 (41.75, 43.35)	44.73 (43.86, 45.60)	52.73 (51.88, 53.57)	< 0.001
**Gender (%)**				< 0.001
Male	55.19 (53.08, 57.29)	52.78 (50.54, 55.01)	40.07 (38.20, 41.97)	
Female	44.81 (42.71, 46.92)	47.22 (44.99, 49.46)	59.93 (58.03, 61.80)	
**Race (%)**				< 0.001
Mexican American	10.13 (6.97, 14.48)	9.65 (6.88, 13.38)	5.10 (3.67, 7.06)	
Other Hispanic	4.97 (3.37, 7.25)	5.30 (3.57, 7.80)	3.52 (2.44, 5.06)	
Non-Hispanic White	63.86 (56.68, 70.48)	68.93 (63.28, 74.07)	81.06 (77.43, 84.24)	
Non-Hispanic Black	14.96 (11.82, 18.75)	10.31 (8.39, 12.61)	5.23 (3.96, 6.88)	
Other race	6.09 (4.82, 7.66)	5.80 (4.19, 7.99)	5.08 (3.95, 6.52)	
**Education (%)**				< 0.001
Less than high school	23.34 (20.95, 25.92)	18.46 (16.26, 20.90)	14.79 (12.65, 17.21)	
High school	25.52 (22.96, 28.25)	24.07 (21.39, 26.97)	21.69 (19.74, 23.79)	
More than high school	51.14 (47.43, 54.84)	57.46 (53.68, 61.16)	63.52 (60.16, 66.75)	
**Marital status (%)**				< 0.001
Married/living with partner	60.31 (57.54, 63.02)	65.10 (61.59, 68.45)	68.30 (65.66, 70.83)	
Widowed/divorced/separated	18.05 (16.45, 19.76)	16.39 (14.80, 18.11)	20.25 (18.49, 22.13)	
Never married	21.64 (19.30, 24.19)	18.51 (15.71, 21.69)	11.45 (9.87, 13.23)	
Poverty income ratio	2.78 (2.66, 2.90)	3.06 (2.94, 3.17)	3.30 (3.17, 3.42)	< 0.001
**Alcohol (%)**				0.011
≥ 12 alcohol drinks per year	80.01 (77.75, 82.10)	77.62 (74.81, 80.19)	73.05 (69.88, 76.00)	
< 12 alcohol drinks per year	19.99 (17.90, 22.25)	22.38 (19.81, 25.19)	26.95 (24.00, 30.12)	
**Smoking (%)**				< 0.001
Current smokers	32.68 (29.84, 35.66)	19.60 (17.73, 21.61)	13.03 (11.33, 14.95)	
Formerly smoked	19.92 (17.96, 22.04)	25.19 (22.75, 27.79)	29.47 (27.45, 31.57)	
Never smoked	47.39 (44.01, 50.80)	55.21 (51.70, 58.67)	57.50 (54.54, 60.41)	
**Physical activity (%)**				0.001
Inactive	53.26 (50.21, 56.28)	52.73 (49.70, 55.74)	57.07 (53.83, 60.26)	
Moderate	6.65 (5.65, 7.81)	7.45 (6.37, 8.69)	9.32 (7.82, 11.06)	
Vigorous	40.09 (36.96, 43.31)	39.82 (37.01, 42.71)	33.61 (30.16, 37.25)	
**BMI (%)**				< 0.001
< 25	35.76 (33.18, 38.42)	29.44 (26.57, 32.48)	28.08 (25.41, 30.93)	
25–30	32.10 (30.12, 34.15)	35.95 (33.33, 38.67)	34.15 (32.40, 35.94)	
≥ 30	32.14 (30.46, 33.87)	34.61 (32.09, 37.21)	37.76 (35.50, 40.08)	
**Hypertension (%)**				0.001
Yes	45.32 (43.25, 47.42)	45.64 (43.20, 48.10)	51.74 (48.24, 55.22)	
No	54.68 (52.58, 56.75)	54.36 (51.90, 56.80)	48.26 (44.78, 51.76)	
**Diabetes (%)**				0.004
Yes	10.44 (8.83, 12.30)	11.44 (10.06, 12.99)	13.92 (12.09, 15.98)	
No	89.56 (87.70, 91.17)	88.56 (87.01, 89.94)	86.08 (84.02, 87.91)	
Cardiovascular disease* (%)				< 0.001
Yes	7.13 (5.94, 8.52)	6.50 (5.59, 7.55)	10.19 (8.66, 11.97)	
No	92.87 (91.48, 94.06)	93.50 (92.45, 94.41)	89.81 (88.03, 91.34)	
**Cancer (%)**				< 0.001
Yes	6.93 (5.85, 8.19)	7.13 (5.84, 8.68)	13.97 (12.52, 15.56)	
No	93.07 (91.81, 94.15)	92.87 (91.32, 94.16)	86.03 (84.44, 87.48)	
PhenoAge (years)	34.40 (33.23, 35.56)	34.98 (33.85, 36.10)	42.47 (41.13, 43.81)	< 0.001
PhenoAgeAccel (years)	−8.15 (−8.80, −7.50)	−9.75 (−10.25, −9.26)	−10.25 (−10.96, −9.55)	0.001
RBC folate (ng/ml)	375.45 (367.27, 383.63)	504.74 (493.75, 515.74)	709.62 (690.52, 728.71)	< 0.001

Data are presented as mean (SD) or *n* (%). *p* < 0.05 indicates statistical significance. RBC, red blood cell; BMI, body mass index; PhenoAge, phenotypic age; PhenoAgeAccel, phenotypic age acceleration. Data were presented as weighted means or percentages (95% confidence intervals). *Cardiovascular disease includes coronary heart disease, angina, congestive heart failure, heart attack, and stroke.

### 3.2 Relationship between RBC folate and PhenoAgeAccel

The associations between RBC folate and PhenoAgeAccel are presented in Table 2. The results indicated that RBC folate had no significant correlation with PhenoAgeAccel in the non-adjusted model (β: 0.0008, 95% CI: −0.0008, 0.0025), the partially adjusted model (β: 0.000895, 95% CI: −0.0009, 0.0026), and the fully adjusted model (β: 0.0005, 95% CI: −0.0012, 0.0022). After RBC folate was classified into tertiles, in the fully adjusted models, participants in the moderate RBC folate level group were found to be 0.7299 years younger (β: −0.7299, 95% CI: −1.2882, −0.2317) than the low RBC folate level group. However, there was no significant change in PhenoAgeAccel in the high RBC folate level group compared to the low RBC folate level group (β: −0.5898, 95% CI: −1.3481, −0.1686). There was no statistically significant trend between RBC folate levels and PhenoAgeAccel (*P* for trend > 0.05).

**TABLE 2:** Associations between RBC folate and PhenoAgeAccel.

	Model 1^a^		Model 2^b^		Model 3^c^	
	β (95% CI)	*P*-value	β (95% CI)	*P*-value	β (95% CI)	*P*-value
Continuous	0.0008 (−0.0008, 0.0025)	0.318	0.0008 (−0.0009, 0.0026)	0.365	0.0005 (−0.0012, 0.0022)	0.572
**Categories**						
Low	Reference		Reference		Reference	
Moderate	−0.9675 (−1.5304, −0.4046)	0.002	−0.8427 (−1.3802, −0.3051)	0.005	−0.7299 (−1.2282, −0.2317)	0.018
High	−0.6295 (−1.4558, 0.1968)	0.146	−0.6447 (−1.4588, 0.1693)	0.134	−0.5898 (−1.3481, 0.1686)	0.162
*P* for trend		0.175		0.152		0.178

RBC, red blood cell; Q, quartile; PhenoAgeAccel, phenotypic age acceleration. *^a^*Model 1: adjusted for no covariates. *^b^*Model 2: adjusted for age, gender, and race. *^c^*Model 3: adjusted for age, gender, race, education, marital status, poverty income ratio, BMI, alcohol, smoking, physical activity, hypertension, diabetes, cardiovascular disease, and cancer.

**FIGURE 2:**
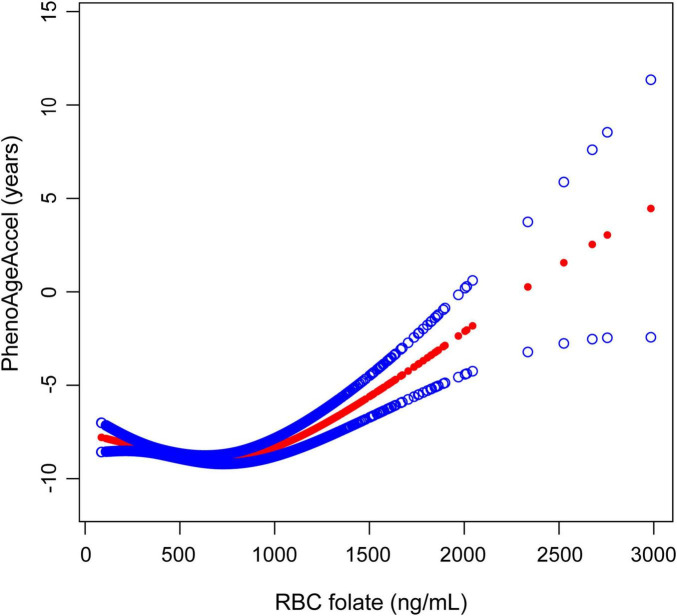
The association between RBC folate and PhenoAgeAccel. The solid red line represents the smooth curve fit between variables, with blue bands representing the 95% CI of the fit. RBC, red blood cell; PhenoAgeAccel, phenotypic age acceleration.

The smooth curve fits model demonstrated a non-linear relationship between RBC folate and PhenoAgeAccel (Figure 2). RBC folate levels exhibited a U-shaped dose-response relationship with PhenoAgeAccel. Threshold effect analysis showed that the inflection point of RBC folate was observed at 732.9 ng/mL (*P* for likelihood ratio test < 0.001). The PhenoAgeAccel decreased by 0.0027 years per 1 ng/mL increase in RBC folate when RBC folate ≤ 732.9 ng/mL (β: −0.0027, 95% CI: −0.0051, −0.0002), and increased by 0.0058 years per 1 ng/mL increase in RBC folate when RBC folate > 732.9 ng/mL (β: 0.0058, 95% CI: 0.0026, 0.0090) (Table 3).

**TABLE 3:** Threshold effect analysis of RBC folate on PhenoAgeAccel.

Outcome	β (95% CI)	*P*-value
One–line linear regression model	0.0005 (−0.0012, 0.0022)	0.572
**Two–piecewise linear regression model**		
RBC folate ≤ 732.9	−0.0027 (−0.0051, −0.0002)	0.035
RBC folate > 732.9	0.0058 (0.0026, 0.0090)	0.003
Log–likelihood ratio test		< 0.001

RBC, red blood cell; PhenoAgeAccel, phenotypic age acceleration.

### 3.3 Subgroup analysis

**FIGURE 3:**
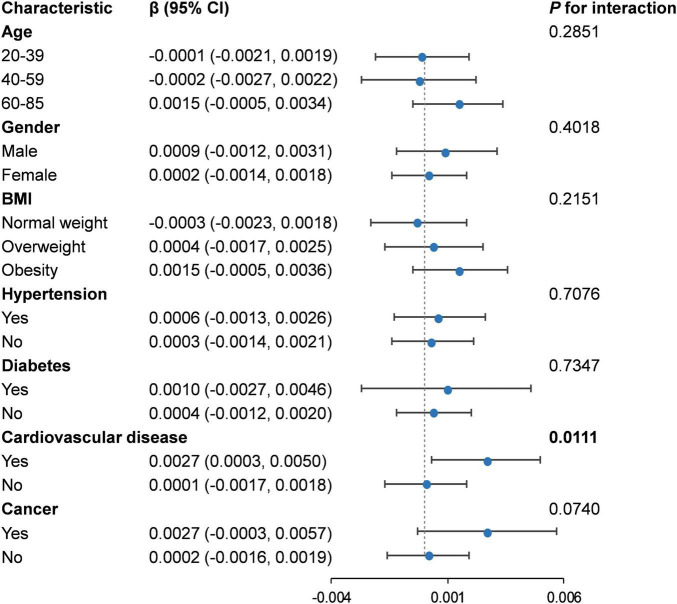
Subgroup analysis for the association between RBC folate and PhenoAgeAccel. RBC, red blood cell; BMI, body mass index.

There was almost no significant difference suggested by the interaction test (*P* for interaction > 0.05) in the association of RBC folate and PhenoAgeAccel among different subgroups (Figure 3). However, an exception was observed in the subgroup of patients with cardiovascular diseases, where a significant positive correlation was identified between RBC folate and PhenoAgeAccel (β: 0.0027, 95% CI: 0.0003, 0.0050) (*P* for interaction = 0.0111) (Supplementary Figure 1).

## 4 Discussion

In this cross-sectional study, we found a U-shaped relationship between RBC folate levels and PhenoAgeAccel in US adults. Notably, we observed an inflection point at 732.9 ng/mL. Below this threshold, RBC folate levels were negatively associated with PhenoAgeAccel, while above this value, the association reversed direction, showing a positive relationship.

Previous studies of folate and various aging indicators have been conducted in different populations, generally suggesting that aging decreases as folate concentrations increase (24–26). However, our findings show a U-shaped association between RBC folate levels and accelerated aging, as measured by PhenoAgeAccel. We speculate that this discrepancy may be due to differences in samples and measures used for assessing aging. Notably, emerging evidence suggests that increased folate levels do not always confer health benefits and may, in fact, adversely affect aging (27–30), supporting our findings. Faux et al. (31) and Zhou et al. (32) observed a U-shaped association in their studies of RBC folate and homocysteine, and RBC folate and the risk of severe aortic arch calcification, respectively. These studies indicate that both folate deficiency and excess can increase the risk of age-related diseases. Another concern observed in our study is that an increase in PhenoAgeAccel was significant in the cardiovascular disease group when RBC folate concentrations exceeded a certain threshold. This finding aligns with previous studies showing increased mortality and heart disease risk in individuals with elevated folate levels (33–35).

Biological aging is driven by complex interactions involving dysregulated cellular homeostasis and biochemical processes (36). For instance, as a key nutrient in one–carbon metabolism, folate provides the methyl donor S-adenosylmethionine for DNA methylation, thus maintaining normal DNA methylation. During aging, the dysregulation of gene expression and genomic instability are key factors in cellular decline and disease. By regulating DNA methylation, folate may slow these age-related changes and positively impact the aging process (37). Besides, folate is involved in various metabolic processes, playing a crucial role in cell proliferation, DNA repair, energy metabolism, amino acid metabolism, and neurotransmitter synthesis (38). Folate deficiency disrupts these processes, leading to homocysteine accumulation, increased oxidative stress, subtelomere hypomethylation, and uracil incorporation, which result in telomere breakage and shortening (39–42). These processes can also reinforce each other, exacerbating the aging process (29). Folate deficiency or impaired folate metabolism may lead to elevated homocysteine levels, which are known to cause endothelial dysfunction (43). Excessive folate levels also present several problems. It has been reported that a single intake of more than 200 μg of folic acid leads to the accumulation of unmetabolized folic acid (UMFA) in circulation (44). UMFA inhibits DNA synthesis, cause abnormal DNA methylation, which can impair vascular function and contribute to endothelial dysfunction, and can induce cytotoxicity in natural killer cells (38, 44). Additionally, high folate levels have been associated with increased oxidative stress and inflammation, which are key drivers of aging and cardiovascular disease (45). These factors may exacerbate the aging process and worsen the prognosis in individuals with CVD, which could explain our findings in the cardiovascular disease group. Excessive folate often masks the hematological and neurological symptoms of vitamin B12 deficiency, leading to delayed diagnosis of related diseases (46). Both folate deficiency and excess may interfere with normal cell replication and survival, affect metabolism, and facilitate the aging process through various pathways, ultimately accelerating aging and increasing disease risk.

To the best of our knowledge, this is the first study to directly evaluate the relationship between RBC folate levels and PhenoAgeAccel. Our findings contribute to understanding the relationship between RBC folate levels and aging, suggesting that keeping RBC folate in an appropriate range is beneficial for health and delaying aging. This is particularly relevant in the context of folic acid fortification. However, the study does have several limitations. First, due to the cross-sectional nature of the NHANES data, causality cannot be confirmed. Therefore, prospective cohort studies in the future are necessary. Second, despite adjusting for multiple confounding factors, unmeasured variables such as folate supplement intake and levels of unmetabolized folic acid could still potentially influence our findings. Future studies should incorporate more comprehensive data on folate metabolism and supplementation to address these limitations. Finally, the data used in this study are derived from the US population and may not be generalizable to other racial or ethnic groups. These limitations highlight the need for further research to explore the underlying mechanisms and optimal folate concentrations.

## 5 Conclusion

In summary, our findings observed that there is a U-shaped relationship between RBC folate levels and PhenoAgeAccel, indicating that both excessively high and low levels can contribute to accelerated aging. Therefore, maintaining an appropriate concentration of folate is of significant importance in the prevention of aging and its associated diseases.

## Data Availability

Publicly available datasets were analyzed in this study. This data can be found here: Centers for Disease Control and Prevention (CDC), National Center for Health Statistics (NCHS), National Health and Nutrition Examination Survey (NHANES), https://wwwn.cdc.gov/nchs/nhanes/default.aspx, NHANES 2007–2008 and NHANES 2009–2010.

## Footnotes

1
https://www.cdc.gov/nchs/nhanes/index.htm

2
http://www.r-project.org

3
http://www.empowerstats.com

## References

[1] World Health Organization. *Ageing and Health.* Geneva: WHO (2022).

[2] Partridge L Deelen J Slagboom P. Facing up to the global challenges of ageing. *Nature.* (2018) 561:45–56. 10.1038/s41586-018-0457-8 30185958

[3] Goldman D Cutler D Rowe J Michaud P Sullivan J Peneva D Substantial health and economic returns from delayed aging may warrant a new focus for medical research. *Health Aff (Millwood).* (2013) 32:1698–705. 10.1377/hlthaff.2013.0052 24101058 PMC3938188

[4] Liu C Hua L Xin Z. Synergistic impact of 25-hydroxyvitamin D concentrations and physical activity on delaying aging. *Redox Biol.* (2024) 73:103188. 10.1016/j.redox.2024.103188 38740004 PMC11103937

[5] Levine M Lu A Quach A Chen B Assimes T Bandinelli S An epigenetic biomarker of aging for lifespan and healthspan. *Aging (Albany NY).* (2018) 10:573–91. 10.18632/aging.101414 29676998 PMC5940111

[6] Xu C Wang J Song Z Deng H Li C. Mediating role of accelerated aging in the association between depression and mortality risk: Findings from NHANES. *Aging Clin Exp Res.* (2024) 36:202. 10.1007/s40520-024-02854-z 39368008 PMC11455804

[7] Liu Z Kuo P Horvath S Crimmins E Ferrucci L Levine M. A new aging measure captures morbidity and mortality risk across diverse subpopulations from NHANES IV: A cohort study. *PLoS Med.* (2018) 15:e1002718. 10.1371/journal.pmed.1002718 30596641 PMC6312200

[8] Verburgh K. Nutrigerontology: Why we need a new scientific discipline to develop diets and guidelines to reduce the risk of aging-related diseases. *Aging Cell.* (2015) 14:17–24. 10.1111/acel.12284 25470422 PMC4326913

[9] López-Otín C Blasco M Partridge L Serrano M Kroemer G. Hallmarks of aging: An expanding universe. *Cell.* (2023) 186:243–78. 10.1016/j.cell.2022.11.001 36599349

[10] Bhargava S Tyagi S. Nutriepigenetic regulation by folate-homocysteine-methionine axis: A review. *Mol Cell Biochem.* (2014) 387:55–61. 10.1007/s11010-013-1869-2 24213682

[11] Paul L. Diet, nutrition and telomere length. *J Nutr Biochem.* (2011) 22:895–901. 10.1016/j.jnutbio.2010.12.001 21429730

[12] Ormazabal A Casado M Molero-Luis M Montoya J Rahman S Aylett S Can folic acid have a role in mitochondrial disorders? *Drug Discov Today.* (2015) 20:1349–54. 10.1016/j.drudis.2015.07.002 26183769

[13] Jones P Lucock M Scarlett C Veysey M Beckett E. Folate and inflammation – links between folate and features of inflammatory conditions. *J Nutr Intermediary Metab.* (2019) 18:100104. 10.1016/j.jnim.2019.100104

[14] Lisboa J Ribeiro M Luna R Lima R Nascimento R Monteiro M Food Intervention with folate reduces TNF-α and interleukin levels in overweight and obese women with the MTHFR C677T polymorphism: A randomized trial. *Nutrients.* (2020) 12:361. 10.3390/nu12020361 32019154 PMC7071147

[15] Boyles A Yetley E Thayer K Coates P. Safe use of high intakes of folic acid: Research challenges and paths forward. *Nutr Rev.* (2016) 74:469–74. 10.1093/nutrit/nuw015 27272334 PMC5009460

[16] Chen Q Huang J Shi X Peng Y Chen A Huang L Associations between dietary B vitamin intakes and cognitive function among elderly individuals: An observational study. *Nutrition.* (2025) 134:112716. 10.1016/j.nut.2025.112716 40056822

[17] Selhub J. Folate, vitamin B12 and vitamin B6 and one carbon metabolism. *J Nutr Health Aging.* (2002) 6:39–42. 11813080

[18] EFSA. Scientific opinion on dietary reference values for folate. *EFSA J.* (2014) 12:3893. 10.2903/j.efsa.2014.3893 PMC13136993 42087961

[19] CDC. *Health NBBNDoLSNCfE. Laboratory Method Files: Whole Blood and Serum Folate (October 2011).* Atlanta: CDC (2011).

[20] XuCSongZWangJLiC. Association of visceral adiposity index with phenotypic age acceleration: Insight from NHANES 1999-2010. *J Nutr Health Aging.* (2024) 28:100323. 10.1016/j.jnha.2024.100323 39067143

[21] Whelton P Carey R Aronow W Casey D Collins K Dennison Himmelfarb C 2017 ACC/AHA/AAPA/ABC/ACPM/AGS/APhA/ASH/ASPC/NMA/PCNA guideline for the prevention, detection, evaluation, and management of high blood pressure in adults: Executive summary: A report of the american college of cardiology/American heart association task force on clinical practice guidelines. *Circulation.* (2018) 138:e426–83. 10.1161/CIR.0000000000000597 30354655

[22] Menke A Casagrande S Geiss L Cowie C. Prevalence of and trends in diabetes among adults in the United States, 1988-2012. *JAMA.* (2015) 314:1021–9. 10.1001/jama.2015.10029 26348752

[23] Song Z Gu H Xu C. Association of the non-high-density lipoprotein cholesterol to high-density lipoprotein cholesterol ratio with non-alcoholic fatty liver disease and hepatic steatosis in United States adults: Insights from NHANES 2017-2020. *Front Nutr.* (2025) 12:1540903. 10.3389/fnut.2025.1540903 40290661 PMC12021641

[24] Nwanaji-Enwerem J Colicino E Gao X Wang C Vokonas P Boyer E Associations of plasma folate and Vitamin B6 with blood DNA methylation age: An analysis of one-carbon metabolites in the VA normative aging study. *J Gerontol A Biol Sci Med Sci.* (2021) 76:760–9. 10.1093/gerona/glaa257 33027507 PMC8355450

[25] Sae-Lee C Corsi S Barrow T Kuhnle G Bollati V Mathers J Dietary intervention modifies DNA methylation age assessed by the epigenetic clock. *Mol Nutr Food Res.* (2018) 62:e1800092. 10.1002/mnfr.201800092 30350398

[26] Tucker L. Serum and dietary folate and vitamin B12 levels account for differences in cellular aging: Evidence based on telomere findings in 5581 U.S. Adults. *Oxid Med Cell Longev.* (2019) 2019:4358717. 10.1155/2019/4358717 31687079 PMC6800923

[27] Choi J Yates Z Veysey M Heo Y Lucock M. Contemporary issues surrounding folic Acid fortification initiatives. *Prev Nutr Food Sci.* (2014) 19:247–60. 10.3746/pnf.2014.19.4.247 25580388 PMC4287316

[28] Chen P Li C Li X Li J Chu R Wang H. Higher dietary folate intake reduces the breast cancer risk: A systematic review and meta-analysis. *Br J Cancer.* (2014) 110:2327–38. 10.1038/bjc.2014.155 24667649 PMC4007237

[29] Moores C Fenech M O’Callaghan N. Telomere dynamics: The influence of folate and DNA methylation. *Ann N Y Acad Sci.* (2011) 1229:76–88. 10.1111/j.1749-6632.2011.06101.x 21793842

[30] Sauer J Mason J Choi S. Too much folate: A risk factor for cancer and cardiovascular disease? *Curr Opin Clin Nutr Metab Care.* (2009) 12:30–6. 10.1097/MCO.0b013e32831cec62 19057184 PMC2790187

[31] Faux N Ellis K Porter L Fowler C Laws S Martins R Homocysteine, vitamin B12, and folic acid levels in Alzheimer’s disease, mild cognitive impairment, and healthy elderly: Baseline characteristics in subjects of the Australian Imaging Biomarker Lifestyle study. *J Alzheimers Dis.* (2011) 27:909–22. 10.3233/JAD-2011-110752 21891867

[32] Zhou L Wen X Peng Y Guo M Zhao L. Red blood cell folate and severe abdominal aortic calcification: Results from the NHANES 2013-2014. *Nutr Metab Cardiovasc Dis.* (2021) 31:186–92. 10.1016/j.numecd.2020.08.020 32988723

[33] Vanderwall C Tangney C Kwasny M Gustashaw K. Examination of circulating folate levels as a reflection of folate intakes among older adult supplement users and nonusers in the National health and nutrition examination survey 2003-2004. *J Acad Nutr Diet.* (2012) 112:285–90. 10.1016/j.jada.2011.10.001 22732462

[34] Peng Y Wang Z. Red blood cell folate concentrations and coronary heart disease prevalence: A cross-sectional study based on 1999-2012 National health and nutrition examination survey. *Nutr Metab Cardiovasc Dis.* (2017) 27:1015–20. 10.1016/j.numecd.2017.07.007 28844321

[35] Peng Y Dong B Wang Z. Serum folate concentrations and all-cause, cardiovascular disease and cancer mortality: A cohort study based on 1999-2010 National Health and nutrition examination survey (NHANES). *Int J Cardiol.* (2016) 219:136–42. 10.1016/j.ijcard.2016.06.024 27323339

[36] Rutledge J Oh H Wyss-Coray T. Measuring biological age using omics data. *Nat Rev Genet.* (2022) 23:715–27. 10.1038/s41576-022-00511-7 35715611 PMC10048602

[37] Choi S Friso S. Modulation of DNA methylation by one-carbon metabolism: A milestone for healthy aging. *Nutr Res Pract.* (2023) 17:597–615. 10.4162/nrp.2023.17.4.597 37529262 PMC10375321

[38] Alnabbat K Fardous A Cabelof D Heydari A. Excessive folic acid mimics folate deficiency in human lymphocytes. *Curr Issues Mol Biol.* (2022) 44:1452–62. 10.3390/cimb44040097 35723355 PMC9164024

[39] Cagnacci A Cannoletta M Xholli A Piacenti I Palma F Palmieri B. Folate administration decreases oxidative status and blood pressure in postmenopausal women. *Eur J Nutr.* (2015) 54:429–35. 10.1007/s00394-014-0726-8 24906471

[40] Joshi R Adhikari S Patro B Chattopadhyay S Mukherjee T. Free radical scavenging behavior of folic acid: Evidence for possible antioxidant activity. *Free Radic Biol Med.* (2001) 30:1390–9. 10.1016/s0891-5849(01)00543-3 11390184

[41] Kawanishi S Oikawa S. Mechanism of telomere shortening by oxidative stress. *Ann N Y Acad Sci.* (2004) 1019:278–84. 10.1196/annals.1297.047 15247029

[42] Fenech M. The role of folic acid and Vitamin B12 in genomic stability of human cells. *Mutat Res.* (2001) 475:57–67. 10.1016/s0027-5107(01)00079-3 11295154

[43] Xu X Wei W Jiang W Song Q Chen Y Li Y Association of folate intake with cardiovascular-disease mortality and all-cause mortality among people at high risk of cardiovascular-disease. *Clin Nutr.* (2022) 41:246–54. 10.1016/j.clnu.2021.11.007 34929527

[44] Kelly P McPartlin J Goggins M Weir D Scott J. Unmetabolized folic acid in serum: Acute studies in subjects consuming fortified food and supplements. *Am J Clin Nutr.* (1997) 65:1790–5. 10.1093/ajcn/65.6.1790 9174474

[45] Chen H Liu S Ji L Wu T Ji Y Zhou Y Folic Acid Supplementation mitigates Alzheimer’s disease by reducing inflammation: A randomized controlled trial. *Mediators Inflamm.* (2016) 2016:5912146. 10.1155/2016/5912146 27340344 PMC4909909

[46] Selhub J Rosenberg I. Excessive folic acid intake and relation to adverse health outcome. *Biochimie.* (2016) 126:71–8. 10.1016/j.biochi.2016.04.010 27131640

