# Supplementary material for: Association between red blood cell folate and accelerated aging in American adults: a cross-sectional study from the national health and nutrition examination survey
Source: Front Nutr. 2025 Jun 19;12:1504441. doi: 10.3389/fnut.2025.1504441 (PMC12224436; doi:10.3389/fnut.2025.1504441)
Supplement: Supplementary file 1 [file Data_Sheet_1.docx]

**Supplementary materials**

**Title:** Association between red bloodcell folate and accelerated aging in American adults: a cross-sectional study from the national health and nutrition examination survey

**Authors:** Jia-ni Wang^1^, Zhen Song^2^, Cheng Xu^3^*, Chong-chao Li^1^*

^1^Institute of Literature in Chinese Medicine, Nanjing University of Chinese Medicine, Nanjing, China;

^2^Yancheng Binhai Hospital of Traditional Chinese Medicine, Yancheng, China;

^3^The First Clinical Medical College, Nanjing University of Chinese Medicine, Nanjing, China

**Correspondence to:** Chong-chao Li, Nanjing University of Chinese Medicine, Nanjing, China (lichongchao@njucm.edu.cn)

Cheng Xu, Nanjing University of Chinese Medicine, Nanjing, China (xucheng@njucm.edu.cn)

**Supplementary Methods 1** Calculations of PhenoAge and PhenoAgeAccel

The resulting final equations for calculating PhenoAge and PhenoAgeAccel in this study are as follows:

$$PhenoAge=141.50+\frac{\ln\left[ -0.00553\times\ln\left( 1-Mortality risk \right) \right]}{0.090165}$$

$$\mathrm{PhenoAgeAccel}=Phenotypic A\mathrm{ge}-Chronological Age$$

Where:

$$Mortality risk=1-exp(\frac{-1.51714\times exp(xb)}{0.0076927})$$

And:

$$xb=-19.907-0.0336\times albumin+0.0095\times Creatinine+0.1953\times Glucose+0.0954\times ln\left( \mathrm{CRP} \right)-0.0120\times Lymphocyte Percent+0.0268\times Mean Cell Volume+0.3306\times Red Cell Distribution Width+0.00188\times Alkaline Phosphatase+0.0554\times White Blood Cell Count+0.0804\times Chronological Age$$

**
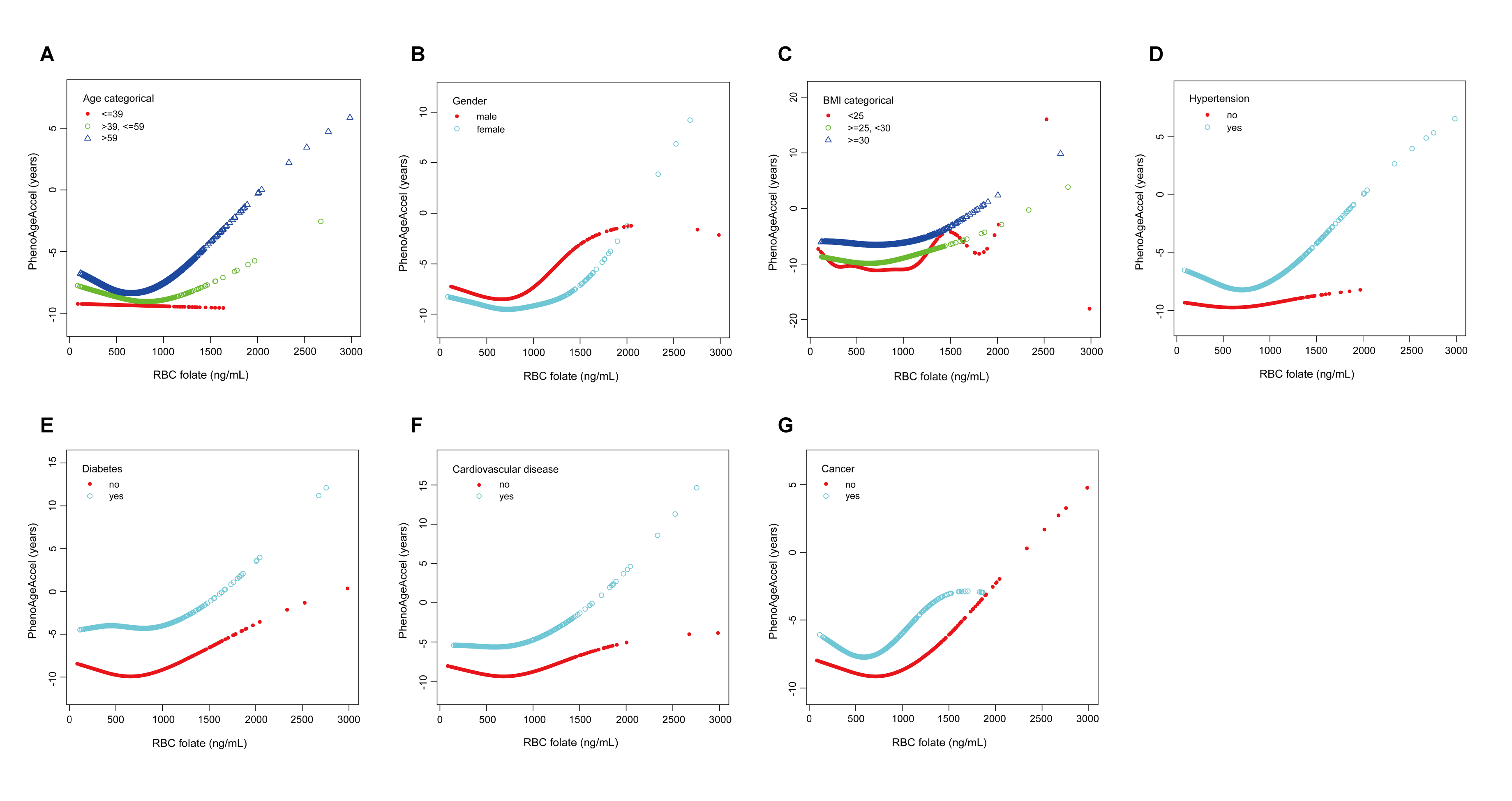
**

**Supplementary Figure S1** The association of RBC folate and PhenoAgeAccel among different stratifications

Solid rad line represents the smooth curve fit between variables. Different colored lines represent each subgroup of results.

Abbreviations: RBC, red blood cell; BMI, body mass index.

1. Age; **B.** Gender; **C.**BMI; **D.** Hypertension; **E.** Diabetes; **F.** Cardiovascular disease; **G.** Cancer
